# Oxygenation time course and neuromuscular fatigue during repeated cycling sprints with bilateral blood flow restriction

**DOI:** 10.14814/phy2.13872

**Published:** 2018-10-07

**Authors:** Sarah J. Willis, Laurent Alvarez, Fabio Borrani, Grégoire P. Millet

**Affiliations:** ^1^ Institute of Sport Sciences Faculty of Biology and Medicine University of Lausanne Lausanne Switzerland

**Keywords:** BFR, central fatigue, occlusion, perfusion

## Abstract

The aim was to evaluate changes in peripheral and cerebral oxygenation, cardiorespiratory, and performance differences, as well as neuromuscular fatigue across multiple levels of blood flow restriction (BFR) during a repeated cycling sprint test to exhaustion (RST). Participants performed three RST (10‐sec maximal sprints with 20‐sec recovery until exhaustion) with measurements of power output and V̇O_2peak_ as well as oxygenation (near‐infrared spectroscopy) of the vastus lateralis and prefrontal cortex. Neuromuscular fatigue was assessed by femoral nerve stimulation to evoke the vastus lateralis. Tests were conducted with proximal lower limb bilateral vascular occlusion at 0%, 45%, and 60% of resting pulse elimination pressure. Total work decreased with BFR (52.5 ± 22.9% at 45%, 68.6 ± 32.6% at 60%, *P *< 0.01 compared with 0%) as V̇O_2peak_ (12.6 ± 9.3% at 45%, 18.2 ± 7.2% at 60%, compared with 0%*, P *< 0.01). Decreased changes in muscle deoxyhemoglobin (∆[HHb]) during sprints were demonstrated at 60% compared to 0% (*P *< 0.001). Changes in total hemoglobin concentrations (∆[tHb]) increased at both 45% and 60% compared with 0% (*P *< 0.001). Cerebral ∆[tHb] increased toward exhaustion (*P *< 0.05). Maximal voluntary contraction (MVC), voluntary activation level (VAL), and root mean square (RMS)/M‐wave ratio decreased at 60% compared with 0% (*P *< 0.001, all). MVC and VAL decreased between 45% and 60% (*P *< 0.05, both). The application of BFR during RST induced greater changes in tissue perfusion (via blood volume, ∆[tHb]) suggesting a possible stimulus for vascular blood flow regulation. Additionally, high‐intensity sprint exercise with partial ischemia may challenge cerebral blood flow regulation and influence local fatigue development due to protection of cerebral function.

## Introduction

Repeated sprinting at maximal intensity with incomplete recoveries (work to rest ratio < 1:4) elicits the early development of fatigue (~33–35% power decrement), therefore impacting performance especially when exercising to exhaustion (Bishop et al. 2004). This type of exercise presents a challenge for both the muscle (accumulation of metabolites) (Spriet et al., 1989) and the motor cortex (i.e., neural drive and altered muscle recruitment) (Ross et al. 2001). The limiting factors at the muscular level encompass (1) muscle excitability due to ionic disturbances (Clausen et al. 1998), (2) energy supply from the phosphocreatine availability, as well as anaerobic glycolysis, and oxidative metabolism as the sprints continue (Gaitanos et al. 1993). The contribution of these factors limits performance and is exaggerated when there are environmental stresses such as altitude (reduced oxygen availability) (Balsom et al. 1994; Brosnan et al. 2000).

When performed in hypoxia, repeated sprinting performance is reduced mainly due to the lower muscle re‐oxygenation during the recovery bouts (Billaut and Buchheit 2013). In addition to the muscular deoxygenation limitations, arterial oxygen saturation, and brain oxygenation are decreased leading to a reduction in motor unit activation (Billaut et al. 2013). Indeed, larger deoxygenation of the prefrontal cortex in acute moderate hypoxia (3800 m) has elicited impaired repeated sprint ability (Smith and Billaut 2010; Willis et al. 2017). It has been suggested that the accumulation of metabolites such as H^+^ ions, due to decreased oxygen delivery to both muscle and brain as well as fatigue during high‐intensity exercise, may trigger sensory feedback to the central nervous system (CNS) via group III and IV afferents, thus indicating a possible explanation for increased central fatigue and reduced power output in hypoxia (Amann et al. 2006; Morales‐Alamo et al. 2015). Therefore, central fatigue may originate from exacerbation of muscle metabolism changes in increased severity hypoxic conditions (Millet et al. 2012). It was recently demonstrated in cerebral tissue that greater changes in total hemoglobin (∆[tHb]), indirectly interpreted as changes in blood volume (Ijichi et al. 2005; Billaut and Buchheit 2013; Faiss et al. 2013; Van Beekvelt et al., 2001), were found in the end of a repeated sprint test to exhaustion regardless of the level of hypoxia (Willis et al. 2017) (indirectly indicated by near‐infrared spectroscopy (NIRS) signals). This finding was likely related to the protection of brain function in low oxygen conditions with a consequential risk of increased hemodynamic brain vessel injury due to high blood flow (Curtelin et al. 2017).

Researchers have demonstrated that severe hypoxia can be created locally in the muscle tissue (ischemia) by applying a blood flow restriction (BFR, also known as vascular occlusion) to the limbs during exercise (Scott et al. 2014). An external application of pressure is sufficient to maintain arterial inflow (partial vascular occlusion) while completely (or near completely) occluding the venous outflow of blood (Kaijser et al. 1990), thus reducing local blood flow. It is currently unknown if this form of local hypoxia (ischemia) can elicit similar and effective changes in blood volume as a stimulus to improve vascularization similar to performing repeated sprints in hypoxia (RSH) (systemic hypoxia). To date, the primary research in the field of BFR has been focused on resistance training and the potential effects on muscle mass and strength (Takarada et al. 2002; Abe et al. 2005, 2006). However, some research has been conducted on cycling and walking (Abe et al. 2010a,b) or swimming (Salem AEEAeaHH, 2011). Several mechanisms were associated with BFR during resistance exercise, from which the primary mechanisms are muscle tension and metabolic stress (metabolite accumulation; P_i_ and decreased pH) (Pearson and Hussain 2015). Furthermore, Manini and Clark (Manini and Clark 2009) suggested that this local accumulation of metabolites, as a result of vascular occlusion, may increase stimulation of group III and IV muscle afferents, which may suggest central fatigue similar to that above from systemic hypoxia (Amann et al. 2006). It is possible that feedback to the CNS from the stimulation of intramuscular pain receptors from metabolic byproducts of muscular contractions are influenced in the presence of BFR; as it has been suggested that the stronger the stimulus and greater rate of peripheral fatigue development, the greater the inhibitory afferent feedback to the CNS leading to decreased central motor drive (Amann and Dempsey 2008). However, there are studies also showing that increased group III and IV muscle afferents have only negligible impact on sprint performance demonstrating a functional reserve for power generation even after exhaustive exercise (Morales‐Alamo et al. 2015; Torres‐Peralta et al. 2015). There are several considerations regarding BFR which include the following parameters: changes in peripheral blood flow, central responses of the cardiovascular system (cardiac output), cerebral oxygenation and blood flow regulation, blood coagulation, oxidative stress levels, muscle damage, and nerve conduction velocity. The efficacy of responses to these parameters depends on the cuff size and pressure, as well as the type of intervention pertaining to the exercise mode, intensity, and duration (Wernbom et al. 2008; Cunniffe et al. 2017). These responses may apply to the use of BFR during aerobic and anaerobic exercise, although it is currently unknown as these exercises are different.

The physiological and neuromuscular responses of repeated sprints to exhaustion (RST) with local BFR (via ischemia) remain unknown. Furthermore, there is a need to investigate which severity of BFR elicits an appropriate physiological response to this stimulus and if BFR could be considered as a form of hypoxic training by challenging the oxygen transport system. Therefore, the aim of this study was to assess the changes in peripheral and cerebral oxygenation and fatigue as well as the physiological responses (power output, oxygen uptake, ventilation, heart rate, rate of perceived exertion) and neuromuscular fatigue with different levels of BFR during RST. It was expected that performance would be limited in restricted blood flow conditions due to both the lower oxygen delivery and the increased perceived exertion and peripheral fatigue of the legs. Furthermore, the hypothesis was that greater changes were elicited in peripheral deoxygenation and total hemoglobin during sprints as the severity of BFR increased. Additionally, the challenge of blood flow regulation near exhaustion would be highlighted by changes in cerebral oxygenation in all conditions, and possibly alter central drive as the severity of BFR increases.

## Methods

### Participants

Eleven healthy and recreationally active volunteers took part of this study (six men and five women; 26.7 ± 4.2 years old, 68.0 ± 14.0 kg, 1.72 ± 0.12 m, 14.1 ± 4.7% body fat). Requirements of participants were to train at least 4‐h per week and to be accustomed to maximal intensity exercise. Participants would be excluded if there were any skeletal or muscular injury in the last three months, pain, or any other medical condition which could compromise the study. Written informed consent was given by participants after being informed of the procedures and risks involved. The experimental protocol was approved by the Ethical Commission for Human Research (CER‐VD 138/15) and performed following the seventh Declaration of Helsinki (2013). Participants were asked to avoid strenuous activity as well as caffeine or alcohol consumption 24 h before each visit. Further, all visits were scheduled at the same time of day for standardization and with at least 48 h between in order to limit fatigue.

### Study design

Participants reported to the laboratory for a total of four sessions (1 familiarization and 3 testing visits) as part of a randomized protocol to assess repeated sprint ability to exhaustion. The testing visits were performed with three different levels of blood flow restriction; no BFR (0%), 45%, and 60% of the pulse elimination pressure (described in detail below). As no previous studies have performed this task, these percentages were chosen based on many pilot testing sessions with repeated sprints to exhaustion across the full range of occlusion levels with critical assessment of set duration, power output, torque factor, pedaling frequency, as well as perceived effort.

### Familiarization

Measurements of anthropometric data (body height, body mass, and skin fold measurement) were collected along with completing the informed consent and health questionnaire [Physical Activity Readiness Questionnaire, PAR‐Q & YOU, (Physiology CSfE, 2002)]. Skin fold measurements were obtained by an experienced technician using the seven‐site formula from the 2010 ACSM guidelines (Medicine ACoS, 2014). The BFR cuff (11 × 85 cm cuff size, 10 × 41 cm bladder size, SC10D Rapid Version Cuff, D.E. Hokansson Inc., Bellevue, WA, USA) was placed on the proximal lower limb for measurement of the pulse elimination pressure. Pulse elimination pressure was measured during seated rest with leg comfortably extended by gradually inflating the cuff until the point at which no more arterial blood flow was detected via Doppler ultrasound (EchoWave II 3.4.4, Telemed Medical Systems, Telemed Ltd. Lithuania, Milano, Italy) of the femoral artery within the adductor canal and was measured two or three times for accuracy, with approximately 2 min between trials (Gualano et al. 2010). Familiarization to the neuromuscular assessment was then conducted including maximal voluntary contractions (MVC), as well as stimulations at different frequencies (100 Hz, 10 Hz, twitch) at rest and during MVC (superimposed 100 Hz) following the protocol described in detail below. Participants were then seated on an electronically braked cycling ergometer (Lode Excalibur Sport Ergometer, Lode B.V., Netherlands) and dimensions were recorded for standardization during subsequent sessions. After a 5‐min warm‐up at 1.5 W·kg^−1^, participants performed two 10‐sec maximal sprints with 3 min of active recovery between with no BFR. Following an additional 5‐min passive recovery, participants were familiarized with the RST with no BFR, as described in detail below. All sprints were performed using the “Wingate mode” from the manufacturer with an individually fixed torque factor of 0.8 Nm·kg^−1^.

### Testing visits

**Figure 1:**
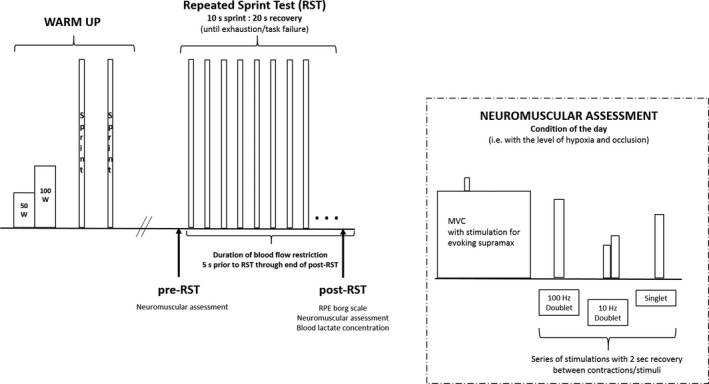
Illustration of the general protocol design including warm‐up and repeated sprint test to exhaustion (RST) with blood flow restriction.

Figure 1 illustrates the protocol of the testing visit. Each session began with a 12‐min warm‐up (6 min at 50 W followed by 6 min at 100 W) at a cadence of 85 rpm. Then two maximal 10‐sec warm‐up sprints were performed (similar to the familiarization visit) with three min of active recovery between sprints. After a 5‐min passive recovery, the measurements for pre‐RST were collected with the BFR cuffs placed bilaterally to the most proximal part of each lower limb and inflated to the pressure of the condition (i.e., no BFR, 45%, or 60%) 1 min prior in order to collect the pre‐RST after the initial kinetic response of blood flow restriction. The cuffs remained inflated throughout the duration of the pre‐RST measurements. For the RST, BFR cuffs were inflated 5 sec before the start and remained inflated during the entire duration of the RST and through the end of the post‐RST measures. The neuromuscular assessment was performed pre‐RST. Participants were then fit with a mask for oxygen uptake measurement and performed the RST. Measurements of pulse oxygen saturation (SpO_2_) and near‐infrared spectroscopy (NIRS) were obtained during the test. Following the completion of the test, the rating of perceived exertion was recorded, followed by the neuromuscular assessment in the chair ergometer (approximately 3‐min post‐RST), and blood lactate concentration (approximately 4‐min post‐RST).

### Repeated sprint test

Participants performed the RST after the warm‐up period, as previously described (Faiss et al. 2013). After pedaling at 20 W with a cadence of 85 rpm for 1 min, with 5 sec before the first sprint, the BFR cuffs were inflated to the test condition (0%, 45%, or 60%), and then participants were given a 3, 2, 1 countdown and began the RST of 10 sec all‐out maximal sprint and 20‐sec active recovery (1:2 work‐to‐rest ratio) until volitional exhaustion or task failure [cadence < 70 rpm, similar to (Faiss et al. 2013)]. The BFR remained until the post‐RST measurements were complete. At the end of each sprint, the ergometer automatically switched to a resistance of 20 W for the recovery. Participants were instructed to perform each sprint maximally, as hard and fast as possible, and to perform as many sprints as possible. A standing position was allowed, though instruction was given to maintain a similar body position for all sprints. Very strong verbal encouragement was given to participants and there was no indication of the number of sprints performed. The first two sprints were controlled to obtain at least 95% of the peak power from the best sprint from the two warm‐up sprints performed to avoid any pacing strategy. Variables of mean power (mean of all sprints, W), number of sprints performed, and total work (kJ) were obtained, in addition to the calculation of fatigue index or percent decrement (S_dec_(%) = [1 – ($\overset{¯}{S}$/S_best_)] × 100), where $\overset{¯}{S}$ corresponds to the mean of all sprints in RST, and S_best_ corresponds to the highest mean power of either of the first two sprints in RST (Glaister et al. 2008).

### Metabolic measurements

Pulmonary gas exchange was measured continuously breath‐by‐breath (Medgraphics CPX, Loma Linda, CA, USA). Oxygen consumption (V̇O_2_), ventilation (V̇_E_), respiratory exchange ratio (RER), and respiratory rate (RR) were computed. The system was calibrated with a 3‐L syringe (M9474, Medikro Oy, Finland) and a calibration was made with ambient air and known gas mixtures of O_2_ (16%) and CO_2_ (5%) prior to each measurement. The highest 30‐sec average of oxygen uptake was obtained. Heart rate was monitored at 1 Hz with a telemetry‐based heart rate monitor (Polar RS400, Kempele, Finland) for analysis during sprints with the maximum value recorded. After the earlobe was cleaned and dried, a lancet was used to take a small droplet of blood (0.2 *μ*L) into a strip for analysis of blood lactate concentration (Lactate Scout, EKF Diagnostics, GmbH, Leipzig, Germany). The S_p_O_2_ was measured at the earlobe with an oximeter (8000Q2 Sensor, Nonin Medical Inc., Amsterdam, The Netherlands), recorded with one sample every 5 sec, and reported as the lowest stable value of the final minute during the RST. Rating of perceived exertion (RPE) was evaluated using the Borg scale (Bigland‐Ritchie et al. 1986; Balsom et al. 1994; Boushel and Piantadosi 2000; Brosnan et al. 2000; Cerretelli and Grassi 2001; Bishop et al. 2004; Amann et al. 2006, 2007, 2015; Amann and Dempsey 2008; Boushel 2010; Billaut and Buchheit 2013; Billaut et al. 2013; Calbet et al. 2015; Blain et al. 2016) as a perception of effort in both the legs and the breathing immediately after the test.

### Near‐infrared spectroscopy measurements

Muscle oxygenation was assessed using the NIRS technique as previously described (Boushel and Piantadosi 2000). The PortaMon and PortaLite devices (Artinis, Zetten, The Netherlands), which include three light source transmitters (each with two wavelengths of 760 and 850 nm) at 30, 35, and 40 mm distance figuto the receiver, were used to measure muscle oxygenation of the vastus lateralis (PortaMon) and of the prefrontal cortex (PortaLite). Devices were placed into a tightly wrapped transparent plastic to avoid humidity and create a waterproof barrier for proper function and signal quality. The PortaMon was placed on the lower third of the vastus lateralis and attached with double sided tape, then wrapped with tension against the leg to reduce movement during exercise. Permanent pen was used to mark the position and images were taken to reproduce the placement in subsequent visits. The PortaLite was attached on the surface of the left prefrontal cortex using double sided tape, and the subject donned a headband to create a dark environment and maintain a stable position of the probe. Measurements included a standard differential pathlength factor of 4.0 for the vastus lateralis as there is a lack of any clear standard value for the quadriceps during cycling sprints (Faiss et al. 2013) and 6.0 for the prefrontal cortex (van der Zee et al. 1992; Amann et al. 2007). Furthermore, the NIRS has demonstrated to have very high reliability regarding muscle oxygen consumption and local skeletal muscle blood flow during low‐ to moderate‐intensity exercise (Lucero et al. 2018). All signals were recorded at the maximum frequency for each device (10 Hz for PortaMon and 50 Hz for PortaLite) and later exported at 10 Hz for further analysis (Oxysoft 3.0.53, Artinis, The Netherlands). For analysis, a 4th‐order low‐pass zero‐phase Butterworth filter (cutoff frequency 0.2 Hz) was applied to reduce artifacts and smooth perturbations in the signal from pedal strokes (Rodriguez et al. 2018). The maximum and minimum was detected automatically for each sprint using deoxyhemoglobin as the parameter to determine the visual starting point of the test. This allowed successive sprint and recovery phases to be identified, and sprint phases to be further analyzed. The change (∆) for each sprint was defined as the difference between maximum and minimum values for each sprint. Delta concentrations of oxyhemoglobin (∆[O_2_Hb]), deoxyhemoglobin (∆[HHb]), total hemoglobin (∆[tHb]), and tissue saturation index (TSI, %) were obtained. Additionally, the analysis was normalized to the duration of the set to exhaustion; that is, percentage of sprints performed (i.e., 20%, 40%, 60%, 80%, 100%), and a linear interpolation was used to calculate values when there was a fractional number of sprints, as each participant performed a different number of sprints in each condition.

### Neuromuscular measurements

To assess neuromuscular fatigue, surface electromyography (EMG) electrodes (Ag/AgCl) of 10mm surface area (Kendall, Covidien, Mansfield, MA, USA) were positioned on the vastus lateralis (VL) of the right upper leg after skin preparation with razor, sandpaper, and alcohol. The placement was marked with permanent pen and images taken to replicate for subsequent tests. In addition, 5 × 10 cm stimulation electrodes (Compex, Ecublens, Switzerland) were placed on the right femoral nerve (inguinal triangle) and at the same place opposite on the mid‐gluteus maximus. Participants were then seated on a custom‐built chair ergometer and adjusted to obtain a 90˚ knee angle, which was recorded and replicated for subsequent measurements. The chair was equipped with a force gauge (Universal Load Cell, VPG Revere transducers, Germany) at the ankle, leg constraint for right leg extension, and hip and shoulder restraints. Stimulation electrodes were connected to a Digitimer (model DS7AH, Hertfordshire, UK). Evoked force (sampling frequency 1000 Hz) as well as EMG signal (2000 Hz) were recorded with the acquisition system (MP150, BIOPAC, Goleta, CA, USA), and analyzed with software (AcqKnowledge, BIOPAC, Goleta, CA, USA). Optimal stimulation intensity was determined at the start of each testing visit. The intensity of stimulation was gradually increased by 20 mA until the amplitude of the resting single twitch and the M‐wave response reached a plateau. To ensure for recruitment of all motor units of the quadriceps, the intensity of the single twitch was increased from the pre‐determined value by 20%. The neuromuscular assessment protocol (as illustrated within Fig. 1) was conducted in the condition of the day beginning with an MVC with superimposed doublet at 100 Hz aimed for delivery at the time of peak force, followed by 100 Hz stimulation at rest (P100), 10 Hz stimulation at rest (P10), and a single twitch at rest. All stimulations were separated by 2 sec. The amplitude (peak to peak) was assessed for each MVC, as well as the evoked forces from the superimposed doublet at 100 Hz, and the resting P100, P10, and single twitch. The ratio of evoked force at low and high frequency (P10/P100) was calculated. The root mean square (RMS) of the raw EMG signal was assessed using a 250 msec epoch of the peak force during MVC, which was normalized by the amplitude of the M‐wave to obtain the ratio RMS/M‐wave. Further, the voluntary activation level (VAL) was calculated using the following formula (Place et al. 2007):

$$\begin{array}{c} \text{VAL}=(1-\begin{array}{c} \frac{\text{superimposed doublet}\times \text{voluntary torque before superimposed doublet}÷\text{maximal torque}}{\text{potentiated resting doublet amplitude}} \end{array})\times 100. \end{array}$$

### Statistical analysis

Evaluation of cardiovascular estimates, blood flow measurements, and neuromuscular assessment was performed using a linear mixed effects analysis of the relationship between condition (0%, 45%, and 60%) and time (pre or post). Fixed effects included condition and time, and participant was set as the random effect. Measurements of oxygenation were also evaluated with a linear mixed model analysis with fixed effects of condition and set duration (20%, 40%, 60%, 80%, 100% of sprints performed) with participant as the random effect. The remaining variables (performance, gas exchange, pulse oxygen saturation, blood lactate, and RPE) were analyzed with a linear mixed model setting condition as the fixed effect and participant as the random effect. Sex differences were not assessed in this study. After inspection of residual plots, there were no obvious deviations from homoscedasticity or normality. Analyses were executed using R (R Core team 2017, Foundation for Statistical Computing, Vienna, Austria) and nlme4 (Pinheiro et al., 2018). The *P* values were set to 0.05 and obtained by likelihood ratio tests of the full model with the effect in question against the model without (control). Least‐squares means for mixed models [library lsmeans, (Lenth 2016)] using the Tukey method were employed to obtain the contrasts. Values are represented as mean ± standard deviation. A prior analysis of the sample size was based on the re‐oxygenation amplitudes observed in the study by Faiss et al. (2013) which indicated a sample of 10 subjects for a statistical power of 0.8 and an alpha risk of 0.05.

## Results

### Repeated sprint test

Table 1 illustrates all performance data. The number of sprints performed to exhaustion decreased by 47.4 ± 25.4% at 45% and 65.8 ± 36.9% at 60% (*P *< 0.01 for both) when compared with the 0% condition. Total work was similarly decreased by 52.5 ± 22.9% at 45% and 68.6 ± 32.6% at 60% (*P *< 0.01 for both). Maximal heart rate was 7.6 ± 8.6% lower (*P* < 0.01) at 60% compared with 0%. Rating of perceived exertion resulted in higher scores in the legs with both 45% and 60% (*P *< 0.05 for both) and lower scores in the breathing at 60% (*P *< 0.05) when compared with the 0% condition. Fatigue index did not result in any significant differences between conditions.

**Table 1:** Performance and respiratory values during repeated sprint test to exhaustion in blood flow restriction conditions of 0%, 45%, and 60%

	0%	45%	60%
Number of sprints	29.8 ± 13.7	13.1 ± 6.5††	7.5 ± 6.4‡
Mean power (W)	543 ± 135	494 ± 127†	530 ± 160
Fatigue index (% decrement)	26.5 ± 7.2	29.1 ± 7.6	23.1 ± 7.7
Total work (kJ)	162 ± 81	67 ± 36††	42 ± 32††
Maximal heart rate (bpm)	185 ± 9	177 ± 20	171 ± 20††
SpO_2_ (%)	93.8 ± 4.5	90.6 ± 11.8	91.9 ± 6.7
Blood lactate (mmol·L^−1^)	9.5 ± 5.2	7.2 ± 2.8	8.6 ± 5.1
V̇O_2_ (L·min^−1^)	2.72 ± 0.59	2.35 ± 0.54†	2.23 ± 0.51††† ‡
RER	1.11 ± 0.07	1.15 ± 0.08	1.13 ± 0.09
V̇_E_ (L·min^−1^)	137 ± 28	133 ± 36	121 ± 35††‡
RR (br·min^−1^)	66.9 ± 5.0	69.0 ± 11.8	64.8 ± 10.5
RPE legs (Borg 6–20)	17.7 ± 2.0	19.5 ± 0.7†	19.5 ± 0.6†
RPE breathing (Borg 6–20)	18.3 ± 1.4	17.7 ± 1.7	15.5 ± 2.5†

Mean ± SD. SpO_2_, pulse oxygen saturation; V̇O_2_, oxygen uptake; RER, respiratory exchange ratio; V̇_E_, minute ventilation; RR, respiratory rate; RPE, rating of perceived exertion.

†††(*P *< 0.001), ††(*P *< 0.01), †(*P *< 0.05) significant main effect on condition, different from 0%. ‡(*P *< 0.05) significant main effect on condition, different from 45%.

### Metabolic responses

Respiratory responses are also presented in Table 1. Peak oxygen uptake was reduced by 12.6 ± 9.3% at 45% (*P *< 0.05) and 18.2 ± 7.2% at 60% (*P *< 0.001) during RST when compared with 0%, and 6.1 ± 6.2% for the difference between the 45% and 60% condition. In addition, minute ventilation was decreased between the 0% and 60% condition (*P *< 0.01) as well as between 45% and 60% (*P *< 0.05). Other variables (RER, RR, S_p_O_2_, and blood lactate) did not result in any significant differences between conditions.

### Peripheral oxygenation

**Figure 2:**
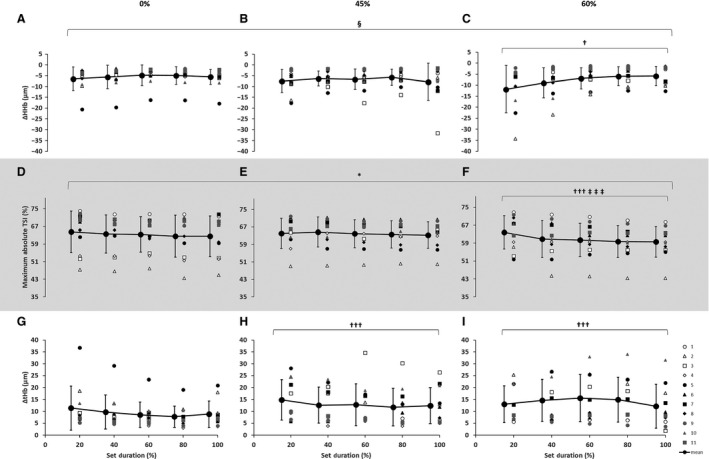
Near‐infrared spectroscopy (NIRS) results representing the average maximum‐minimum delta (∆) value during the percentage of sprints completed to exhaustion for the individual response of the vastus lateralis with blood flow restriction conditions for ∆[HHb] during (A) 0%, (B) 45%, and (C) 60%; for the maximum absolute TSI during (D) 0%, (E) 45%, and (F) 60%; for ∆[tHb] during (G) 0%, (H) 45%, and (I) 60%, respectively. Mean ± SD. †††(*P *< 0.001), †(*P *< 0.05) significant main effect on condition, different from 0%; ‡‡‡(*P *< 0.001) significant main effect on condition, different from 45%; §(*P *< 0.05) significant main effect on set duration, difference between 20% and 80%; * (*P *< 0.05) significant main effect on set duration, difference between 20% and 100%.

As seen in Figure 2, there was a main effect of condition for the vastus lateralis demonstrating lower ∆[HHb] at 60% (*P *< 0.001) compared with 0%. No interactions were present. Absolute maximal TSI values (Fig. 2) demonstrated a main effect of condition with lower values at 60% when compared with both 0% (*P *< 0.001) and 45% (*P *< 0.001), and also a main effect of set duration near exhaustion (*P *< 0.05). Additionally, there were greater ∆[tHb] at both 45% and 60% (*P *< 0.001) compared with 0% (as seen in Fig. 2). There was also a main effect of set duration (20%, 40%, 60%, 80%, 100%) with decreased concentrations of ∆[HHb] and TSI throughout the test (*P *< 0.05). The ∆[O_2_Hb] was greater at 60% when compared with the 0% condition (*P *< 0.05).

### Cerebral oxygenation

**Figure 3:**
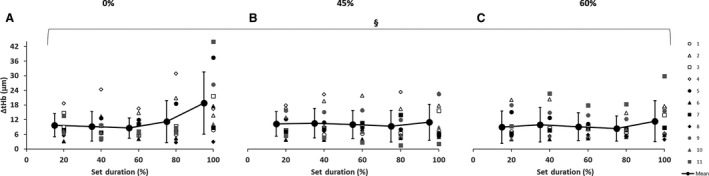
Near‐infrared spectroscopy (NIRS) results representing the average maximum‐minimum delta (∆) value during the percentage of sprints completed to exhaustion for the individual response of the prefrontal cortex with blood flow restriction conditions for ∆[tHb] during (A) 0%, (B) 45%, and (C) 60%. Mean ± SD. §(*P *< 0.05) significant main effect on set duration, difference between 60% and 100%.

In the prefrontal cortex, there was a main effect of set duration with increased ∆[tHb] (Fig. 3) near exhaustion when compared to the beginning of the set. Additionally, the ∆[O_2_Hb] was greater at 100% compared with 60% of set duration (*P *< 0.05). There were no significant differences with ∆[HHb] as well as no interactions.

### Neuromuscular assessment

There were main effects of time (pre‐post) resulting in lower values post‐RST for MVC (*P *< 0.001), VAL (*P *< 0.001), and P10/P100 (*P *< 0.001). There were main effects of condition for MVC, VAL, and RMS/M‐wave all decreasing at the 60% condition when compared with 0% (*P *< 0.001 for all, respectively). Additionally, MVC and VAL indicated a condition main effect decrease between 45% and 60% conditions (*P *< 0.05 for both, respectively), as well as an interaction between condition and time (see Table 2).

**Table 2:** Average neuromuscular responses pre and post repeated sprint test (RST) in conditions of 0%, 45%, and 60% blood flow restriction

	0%	45%	60%	Time main effect	Condition main effect	*P*, interaction
MVC (N)						
Pre	279 ± 140	268 ± 128	263 ± 124		††† ‡	*P *= 0.002
Post	255 ± 114	202 ± 79^3^	138 ± 82^1^^,^ 2 ^,^ 3	***		*F* = 6.9
VAL (%)						
Pre	86 ± 11	86 ± 9	86 ± 10		††† ‡	*P *< 0.001
Post	88 ± 8	81 ± 8	72 ± 13^1^^,^ 2 ^,^ 3	***		*F* = 10.1
P10/P100						
Pre	0.91 ± 0.10	0.92 ± 0.13	0.90 ± 0.14			*P *> 0.05, NS
Post	0.63 ± 0.16	0.68 ± 0.19	0.63 ± 0.18	***		
RMS/M‐wave						
Pre	0.059 ± 0.048	0.059 ± 0.026	0.058 ± 0.033		†††	*P *> 0.05, NS
Post	0.064 ± 0.034	0.054 ± 0.021	0.037 ± 0.027			

Mean ± SD. Cardiovascular measures were obtained at rest prior to RST (pre‐), and at 1‐min post‐RST. Neuromuscular measurements were conducted at rest prior to RST (pre‐), and at approximately 3‐min post‐RST.

MVC, maximal voluntary contraction; VAL, voluntary activation level; P10/P100, ratio of resting stimulations at 10 Hz over stimulation at 100 Hz; RMS/M‐wave, root mean square normalized with the M‐wave.

*** (*P *< 0.001) significant main effect on time, different from 0%.

††† (*P *< 0.001) significant main effect on condition, difference between 0% and 60%.

‡ (*P *< 0.05) significant main effect on condition, difference between 45% and 60%.

^1^ (*P *< 0.05) significantly different from 0%.

^2^ (*P *< 0.05) significantly different from 45%.

^3^(*P *< 0.05) significantly different from pre.

## Discussion

The study's main findings were that (1) performance (number of sprints and total work) decreased with increased BFR, which was concomitant with a decreased peak oxygen consumption; (2) muscle blood volume changes (∆[tHb]) increased with both levels of BFR while smaller changes in peripheral deoxygenation (∆[HHb]) were demonstrated with the 60% condition; (3) changes in cerebral blood volume (∆[tHb]) increased near exhaustion no matter the condition; and (4) the BFR conditions induced large decrements of MVC, VAL, and RMS/M‐wave.

### Performance impairment

As expected, repeated sprint performance declined as the BFR severity increased. The number of sprints and total work decreased to a large extent (~ – 47% and 69% at 45% and 60%, respectively) with occlusion. This was combined with a decreased peak oxygen consumption of up to 18% in the 60% condition. The decreased oxygen consumption and minute ventilation along with decreased maximal heart rate and lower RPE for breathing seem to be related to the reduced set duration in the 60% condition, since as BFR increased in severity, participants were not able to continue to exhaust the cardiovascular and respiratory systems. Rather, participants were limited mainly at the peripheral level as shown by the increased RPE and decreased P10/P100 with BFR. These results were expected, as it is known that peripheral afferent neuronal activity may be stimulated by anabolic factors which are expressed to a larger extent in the presence of BFR (Scott et al. 2014; Pearson and Hussain 2015).

### Peripheral responses

Smaller changes in vastus lateralis deoxygenation (∆[HHb]) was found at 60% when compared with the 0% and 45% conditions (Fig. 2). A plateau in ∆[HHb] has been previously suggested as an indication of maximal skeletal muscle oxygen extraction (Grassi et al. 1999). It is important for interpretation that the method for calculation of change is not the same between previous reports and this study, as this study represents the maximum‐minimum of each sprint performed. For clarity, lower ∆[HHb] does not mean that [HHb] was lower, as the starting value in each sprint for [HHb] was increasing in this study corresponding to power output and exercise intensity. In any case, the current result indicated nearly no change from maximum to minimum in ∆[HHb] or in TSI during sprints at exhaustion no matter the condition, which may support a plateau of ∆[HHb] at the end of exercise. Since Calbet et al. (2015) demonstrated that oxygen extraction measured directly in the leg does not plateau, the present results likely suggest that ∆[HHb] may not be a valid measure of oxygen extraction. Further research is advised for assessment of oxygen extraction during whole body exercise with BFR. It has been shown that oxygenation kinetics may be more reliant on the metabolism of the muscle at intensities above the respiratory compensation point rather than related to oxygen delivery (Cerretelli and Grassi 2001). Indeed, this may be the case in this study, as a change in the metabolic state of the muscle (acidosis and metabolite accumulation) has been previously discussed with BFR conditions (Pearson and Hussain 2015). In addition, the combination of high‐intensity exercise (such as RST) and the severity of BFR would possibly increase oxygen extraction. Furthermore, it remains unknown whether there may be a “reserve” of oxygen extraction, as recently demonstrated when completely occluding blood flow immediately after an incremental ramp test (Morales‐Alamo et al. 2015). Additionally, the present results demonstrated lower absolute muscle TSI in the 60% condition (Fig. 2), as well as a larger decrease near exhaustion no matter the condition.

This study has shown lower oxygen consumption across all BFR conditions and lower maximal heart rate during the 60% condition (Table 1), as well as lower TSI (Fig. 2). This suggests reduced convection likely due to the shorter RST duration. It is well‐known that by increasing oxygen extraction, the oxygen utilization is improved thereby maintaining the oxygen transfer for continuation of exercise even though oxygen delivery may be compromised (Granger and Shepherd 1973). Thus, as blood flow is reduced (via BFR conditions), there could be increased oxygen extraction in order for performance to continue.

In this study, there were increased changes in [tHb], interpreted as changes in tissue blood volume (Ijichi et al. 2005; Van Beekvelt et al., 2001), as the sum of changes in both oxyhemoglobin and deoxyhemoglobin, in both 45% and 60% conditions, when compared with the control. With BFR, the vein is nearly completely occluded, thus increasing the local blood volume and the venous resistance due to reduced venous return. It is important to consider globally that tissue perfusion (volume of blood per unit of time) also involves the amount of active muscle mass. This circulatory adaptation results in more active muscle vascular beds mediated by the sympathetic nervous system to regulate blood flow via the muscle metaboreflex (Clausen 1977; Boushel 2010). Furthermore, an important consideration during intense maximal exercise (large mass of active muscle) is the rise in total vascular conductance which can take over the capacity of the body to raise cardiac output for the amount of work being performed, thus causing the active muscle to vasoconstrict in order to maintain blood pressure. These changes detected in [tHb] may thus create a situation for the body to overcome in order to continue exercise, and therefore, possibly a stimulus for the use of BFR training as a way to induce fluctuations in blood volume (i.e., tissue perfusion) to elicit vascular adaptation.

Peripheral fatigue (P10/P100) in this study was significantly lower during the post‐RST compared with pre‐ no matter the condition. This demonstrated that, similar to previous research, peripheral deficits occurred in all conditions which may indicate an impairment of sarcolemmal excitability and/or neuromuscular propagation (Jubeau et al. 2017). However, it is difficult to interpret the changes of P10/P100 due to low reliability on postexercise force parameters (Doyle‐Baker et al. 2018).

### Cerebral responses

One of the interesting results of this study is that cerebral blood volume (∆[tHb]) increased near exhaustion in all conditions (Fig. 3). This may be due to a neural‐vascular regulatory coupling that increases cerebral blood flow in order to maintain oxygen delivery (Curtelin et al. 2017). This mechanism is partly related to increases in mean arterial blood pressure and ventilation (Curtelin et al. 2017). With increased blood pressure there is an autoregulation challenge with a risk of hyperperfusion in the brain (Curtelin et al. 2017). The present data suggest that changes in cerebral blood volume may contribute to exercise cessation at exhaustion.

Group III and IV muscle afferents are important factors in the development of fatigue as they provide feedback to the CNS regarding hemodynamic responses (circulatory regulation and oxygen delivery) and by projecting an inhibitory effect on the spinal motoneurons which decreases the muscle activation level and elicits central fatigue leading to task failure (Amann and Calbet 2008; Amann et al. 2015; Blain et al. 2016). These sensory neurons are projected during systemic hypoxia (insufficient oxygen delivery and/or low brain oxygenation combined with increased ventilation), thus inducing central fatigue which effects exhaustive endurance performance (Amann and Calbet 2008; Amann et al. 2015). However, it is disputed that afferent feedbacks from skeletal muscle, heart, and lungs contribute to perceived effort during exercise (Marcora, 2009; Marcora, 2010). Furthermore, an influence of psychological factors likely contributes to the magnitude of central motor output during exercise performance (Marcora, 2010). Furthermore, muscle afferents of groups III and IV are also involved in the connection of nociceptors (sensory neuron that respond to damaging or potentially damaging stimuli) to the brain. Therefore, nociceptive afferent feedback is likely involved in goal‐oriented behavior [motivation, (Schultz et al. 1998)] and motor processing (Chaudhuri and Behan 2000). Therefore, as this study demonstrated reduced responses of the central parameters (VAL, RMS/M‐wave) during BFR, it is possible that BFR conditions may also impact the supraspinal fatigue component of central fatigue.

Nevertheless, it must be considered that the NIRS measurements are indirect and should be interpreted with caution. Additionally, the NIRS signal is unable to detect differences between hemoglobin and myoglobin content. Total hemoglobin was not constant and is therefore not suggested as an indication of oxygenation (Grassi et al. 1999), rather the changes in concentration of tHb were regarded as changes in blood volume, and altogether should be interpreted with caution. The method of assessing neuromuscular fatigue was limited due to the delay of measurement of post‐RST (initial assessment followed by moving to the chair ergometer). Since previous research has shown significant recovery in skeletal muscle function within the first 2 min after exercise (Froyd et al. 2013), the analysis of these current measures likely results in a range of about 10–20% variability (Place et al. 2007; Doyle‐Baker et al. 2018). However, it was reported that vascular occlusion prevents the recovery of the MVC and VAL (Bigland‐Ritchie et al. 1986; Gandevia et al. 1996). Even if recent researches using ischemia immediately at the end of exercise have shown partial restoration of power output (Morales‐Alamo et al. 2015; Torres‐Peralta et al. 2015), the differing protocols and intensities of BFR, as well as differing means to measure fatigue warrant further investigation during continuous BFR conditions, as similar to this study. Together, it can be suggested that exhaustive exercise depends more on central than peripheral considerations. The current data also presented an increase in VAL from pre to post measurement, likely explained by a few participants not performing a true MVC possibly due to preconceived notion of stimulation. Although these measurements are problematic in allowing possible recovery of neuromuscular function, most studies have underestimated the extent of fatigue development during dynamic exercise with large muscle mass (Doyle‐Baker et al. 2018), especially regarding central fatigue. Further investigation is required as there may be an influence from the kinetic response of the blood flow restricted environment in the present results due to the cuff inflation. In addition, no measurement was obtained for cerebral blood flow or blood pressure from this study and it is therefore not possible to make further interpretation and further research is warranted, particularly in the evaluation of oxygen extraction and blood flow using the direct invasive methods of Calbet et al. (2015) during BFR conditions.

It was concluded in blood flow restriction conditions that convective oxygen delivery was impaired (lower oxygen consumption) and there were peripheral limitations (peripheral fatigue and perceived exertion), as well as notable decreases in performance. Smaller changes in [HHb] and reduced levels of TSI suggested that oxygen delivery is limited during conditions with high levels of BFR. Furthermore, greater local blood volume (∆[tHb]) was present in both 45 and 60% BFR conditions likely because of increased vascular resistance due to reduced venous return, and therefore may induce a challenge for the vascular system to regulate blood flow (influence of muscle metaboreflex). Additionally, increased cerebral blood volume (∆[tHb]) near exhaustion likely demonstrated the effect of repeated sprints on the regulation of cerebral blood flow for maintenance of oxygen delivery. The BFR conditions in this study combined with exhaustive repeated sprint exercise has probably inhibited spinal output via impacted muscle afferents and elicits central fatigue (altered central drive) possibly similar to systemic hypoxia conditions. Further research is suggested to understand if BFR during repeated sprint exercise can be used as a hypoxic stimulus to benefit performance and clinical health (i.e., vascular diseases) by way of challenging the vascular system for improved oxygen transport to tissues.

## Conflict of Interest

None declared.
